# Use of Digital Health Technologies by Older US Adults

**DOI:** 10.1001/jamanetworkopen.2024.54727

**Published:** 2025-01-15

**Authors:** Cornelius A. James, Tanima Basu, Brahmajee K. Nallamothu, Jeffery T. Kullgren

**Affiliations:** 1 Division of General Medicine, Department of Internal Medicine, University of Michigan, Ann Arbor; 2 Division of General Pediatrics, Department of Pediatrics, University of Michigan, Ann Arbor; 3 Department of Learning Health Sciences, University of Michigan, Ann Arbor; 4 Institute for Health Policy and Innovation, University of Michigan, Ann Arbor; 5 Division of Cardiovascular Medicine, Department of Internal Medicine, University of Michigan, Ann Arbor; 6 Center for Clinical Management Research, Veteran Affairs Ann Arbor Healthcare System, Ann Arbor; 7 Department of Health Management and Policy, University of Michigan, Ann Arbor

## Abstract

This survey study estimated digital health technology (DHT) use and evaluated the factors associated with DHT use by US adults aged 50 to 80 years.

## Introduction

Use of digital health technologies (DHT) has increased in recent years,^1^ accelerated by the COVID-19 pandemic.^2^ Contemporary estimation of older adults’ use of DHT is largely unknown. This study estimated DHT use and evaluated the factors associated with DHT use by older US adults.

## Methods

This survey study followed the STROBE reporting guideline. The institutional review board of the University of Michigan deemed this study exempt and waived the requirement for informed consent because the data were deidentified and publicly available, in accordance with 45 CFR §46. Data were collected through an internet and phone survey in August 2021 as part of the National Poll on Healthy Aging, a recurring survey of adults aged 50 to 80 years. Respondents were randomly selected from the NORC AmeriSpeak panel, a nationally representative probability panel of US households.

Participants provided responses about prior or current use of DHTs and devices that enable use of DHTs. DHTs included patient portals, telehealth encounters, and mobile health (mHealth) applications. Devices included desktop or laptop computers, smartphones, tablets, smartwatches, and fitness trackers. The primary outcome was any use of DHTs. Multivariable logistic regression was used to estimate associations between respondent characteristics (eg, age, sex, self-reported race and ethnicity [queried in the survey and combined into a variable that represented the 2 characteristics], marital status, education, income, type of dwelling, insurance, self-reported physical health, and self-reported mental health) and use of DHTs. The results of the logistic regression model were expressed as adjusted odds ratios (aORs) with 95% CIs, and 2-sided *P* < .05 was considered statistically significant. All statistics were reported as population-level estimates using survey weights designed to reflect population figures from the US Census Bureau. Data were analyzed using SAS version 14.2 (SAS Institute) from February to August 2024.

## Results

A total of 2110 individuals participated in the survey. In weighted analyses, the mean (SD) age of US adults was 63.7 (10.4) years. Among those using a device (95.6%), 53.0% (95% CI, 50.2%-55.7%) were female, 10.8% (95% CI, 9.0%-12.6%) were non-Hispanic Black, 12.2% (95% CI, 10.2%-14.0%) were Hispanic, and 70.1% (95% CI, 67.4%-72.8%) were non-Hispanic White (Table 1). Regarding DHT use (81.4%), 64.5% (95% CI, 61.8%-67.3%) used a patient portal, 49.1% (95% CI, 46.3%-51.8%) used telehealth, and 44% (95% CI, 41.3%-46.7%) used an mHealth application (Table 2).

**Table 1.** Device and Digital Health Technology Use by Older Adults

Characteristic	% (95% CI)		Digital health technology use, aOR (95% CI)^a^
	Device use (n = 2050)^a^	Digital health technology use (n = 1786)^a^	
Overall	95.6 (94.1-97.0)	81.4 (78.9-83.8)	NC
Age, y			
50-59	41.4 (38.6-44.1)	41.4 (38.4-44.3)	2.41 (1.4-4.04)
60-69	34.3 (31.7-36.9	34.8 (32.0-37.5)	1.68 (1.07-2.64)
70-80	24.3 (21.9-26.5)	23.8 (21.3-26.2)	1 [Reference]
Sex			
Male	47 (44.2-49.7)	46.6 (43.6-49.5)	1 [Reference]
Female	53 (50.2-55.7)	53.4 (50.4-56.3)	1.53 (1.06-2.21)
Self-reported race and ethnicity^b^			
Black, Non-Hispanic	10.8 (9-12.6)	11.4 (9.4-13.2)	2.07 (1.02-4.2)
Hispanic	12.2 (10.2-14.0)	11.9 (9.9-13.9)	1.07 (0.6-1.89)
White, Non-Hispanic	70.1 (67.4-72.8)	70.2 (67.4-73.0)	1 [Reference]
Other^c^	6.9 (5.2-8.5)	6.5 (4.4-8.1)	0.56 (0.29-1.07)
Marital status			
Married/living with partner	61.8 (59.0-64.4)	63.2 (60.3-65.9)	1 [Reference]
Widowed	6.8 (5.3-8.2)	6.8 (5.2-8.3)	1.26 (0.61-2.61)
Divorced/separated	20.8 (18.5-23.0)	19.9 (17.5-22.2)	0.71 (0.46-1.1)
Never married	10.6 (8.9-12.2)	10.1 (8.4-11.7)	0.76 (0.43-1.35)
Education			
Less than high school	7.5% (5.2-9.6)	6.9 (4.6-9.1)	1 [Reference]
High school graduate or equivalent	30.1 (27.2-32.8)	26.9 (23.9-29.7)	1.11 (0.55-2.26)
Vocational/tech school/some college/associates	26.3 (24.3-28.2)	27 (24.9-29.2)	2.49 (1.24-5.01)
Bachelor’s/professional degree	36.1 (33.6-38.7)	39.2 (36.3-41.9)	3.53 (1.64-7.59)
Annual income			
<$30 000	21 (18.5-23.4)	18.3 (15.8-20.6)	1 [Reference]
$30 000 to ≤$60 000	26.2 (23.7-28.6)	25.8 (23.1-28.3)	1.98 (1.22-3.22)
≥$60 000	52.9 (49.9-55.5)	55.9 (53.0-58.8)	2.85 (1.64-4.96)
Metropolitan area^d^			
Yes	82 (79.7-84.1)	84.3 (82.1-86.3)	1 [Reference]
No	18.0 (15.8-20.2)	15.7 (13.6-17.8)	0.56 (0.37-0.84)
Insurance			
Medicare only	33 (30.3-35.5)	33.6 (30.7-36.3)	1 [Reference]
Multiple (other than VA/Tricare)	16.9 (14.7-18.9)	16.9 (14.7-19.1)	0.77 (0.48-1.25)
Private insurance	39.9 (37.1-42.5)	40.2 (37.3-43.1)	0.52 (0.31-0.87)
VA/Tricare and other	6.3 (5.0-7.6)	7.1 (5.5-8.5)	1.21 (0.56-2.63)
None	3.9 (2.6-5.1)	2.2 (1.3-3.1)	0.16 (0.07-0.38)
**Self-reported health**			
Excellent	6.3 (5.0-7.6)	6.6 (5.1-7.9)	1 [Reference]
Very good	35.7 (33.0-38.2)	35.7 (32.9-38.4)	1.34 (0.64-2.79)
Good	39 (36.4-41.7)	39.7 (36.8-42.6)	1.79 (0.82-3.91)
Fair	15.4 (13.2-17.4)	14.5 (12.4-16.5)	1.58 (0.64-3.93)
Poor	3.5 (2.2-4.6)	3.4 (2.8-4.7)	1.46 (0.49-4.37)
Do not know or skipped^e^	0.08 (0.1-0.1)	0.1 (0.0-0.3)	NC
**Self-reported mental health**			
Excellent	20.4 (18.2-22.6)	20.8 (18.4-23.1)	1 [Reference]
Very good	39.9 (37.1-42.5)	39.1 (36.3-41.9)	0.74 (0.46-1.18)
Good	26.2 (23.7-28.6)	26 (23.3-28.5)	0.88 (0.5-1.54)
Fair	9.7 (7.9-11.4)	9.8 (7.9-11.5)	1.03 (0.49-2.17)
Poor	1.3 (0.7-1.7)	1.4 (0.7-1.9)	0.57 (0.14-2.25)
Do not know or skipped^e^	2.5 (1.6-3.5)	2.9 (1.8-3.9)	3.46 (0.51-23.33)

Abbreviations: aOR, adjusted odds ratio; NC, not calculated; VA, Veterans Administration.

^a^ Weighted.

^b^ Self-reported from options included in the survey.

^c^ Includes individuals who self-reported non-Hispanic Asian, non-Hispanic Pacific Islander, non-Hispanic other, or non-Hispanic 2 or more races for their racial and ethnic identity. Non-Hispanic other includes individuals who did not report Hispanic ethnicity and did not identify with any of the other categories for race.

^d^ Metropolitan statistical area that includes a county or group of counties with at least 1 urbanized area with a population of at least 50 000 and adjacent counties with economic ties to the central area.

^e^ Answered question “do not know” or skipped the question on the web-based survey.

**Table 2.** Use of Specific Digital Health Modalities by Older Adults^a^

Modality	Any use, % (95% CI)	Current use, % (95% CI)
Patient portal	64.5 (61.8-67.3)	NC
Telehealth	49.1 (46.3-51.8)	NC
mHealth application (overall)	44 (41.3-46.7)	27.8 (25.4-30.2)
Diet	21.4 (19.2-23.6)	7.4 (6.1-8.6)
Exercise	33.8 (31.2-36.3)	21.7 (19.5-23.8)
Weight	20.1 (18-22.2)	8.2 (6.8-9.6)
Sleep	16.7 (14.7-18.7)	9.4 (7.8-10.9)
Mental health	4.6 (3.6-5.7)	2 (1.4-2.7)
Blood pressure	9.2 (7.7-10.7)	6.4 (5.1-7.6)
Meditation	8 (6.7-9.3)	3.7 (2.8-4.6)
Other	7.2 (5.8-8.7)	4.7 (3.6-5.8)

Abbreviations: mHealth, mobile health; NC, not calculated.

^a^ Weighted.

Females were more likely to use DHTs (aOR, 1.53 [95% CI, 1.06-2.21]). In addition, younger age (50-59 years: aOR, 2.41 [95% CI, 1.4-4.04]; 60-69 years: aOR, 1.68 [95% CI, 1.07-2.64]), non-Hispanic Black race and ethnicity (aOR 2.07 [95% CI, 1.02-4.2]), higher education level (vocational/technical school/some college/associates: aOR, 2.49 [95% CI, 1.24-5.01]; bachelor’s/professional degree: aOR, 3.53 [95% CI, 1.64-7.59]), higher income ($30 000 to <$60 000: aOR, 1.98 [95% CI, 1.22-3.22]; ≥$60 000: aOR, 2.85 [95% CI, 1.64-4.96]), metropolitan dwelling (nonmetropolitan dwelling: aOR, 0.56 [95% CI, 0.37-0.84]), and type of insurance (private insurance: aOR, 0.52 [95% CI, 0.31-0.87]; no insurance: aOR, 0.16 [95% CI, 0.07-0.38]) were associated with DHT use (Table 1).

## Discussion

Older adults use various types of DHT, and they use patient portals most often. Although some older adults have unique physical and cognitive needs that can affect the utility and usability of DHTs,^3^ in aggregate they share some predictors of DHT use with younger adults.^2,4^ Additionally, our study showed a statistically significant association between non-Hispanic Black race and ethnicity and DHT use. Various factors may contribute to this finding, including medical mistrust among Black patients leading to decreased engagement with the health system and increased reliance on DHTs.^5^

Study limitations include use of self-reported data, and we did not evaluate some DHTs (eg, internet searches, social media) reported in other studies.^2,4^^,^ Additionally, reported use of technology does not indicate independence because caregivers may assist with technology use. This study highlights the need to carefully consider the unique characteristics of older adults when developing and deploying DHTs to avoid worsening the digital divide.^6^

## Notes

Supplement.
Data Sharing Statement
