# Supplementary material for: Use of Digital Health Technologies by Older US Adults
Source: JAMA Netw Open. 2025 Jan 15;8(1):e2454727. doi: 10.1001/jamanetworkopen.2024.54727 (PMC11736496; doi:10.1001/jamanetworkopen.2024.54727)
Supplement: Supplement. — Data Sharing Statement [file jamanetwopen-e2454727-s001.pdf]

Data Sharing Statement

James. Use of Digital Health Technologies by Older US Adults. *JAMA Netw Open*. Published January 15, 2025.
doi:10.1001/jamanetworkopen.2024.54727

Data
Data available: Yes
Data types: Deidentified participant data
How to access data: https://www.openicpsr.org/openicpsr/search/studies?start=0&ARCHIVE=openicpsr&sort=score%20desc%2CDATEUPDATED%20desc&rows=25&q=national%20poll%20on%20healthy%20aging
When available: With publication

Supporting Documents
Document types: None

Additional Information
Who can access the data: Anyone requesting the data may access it freely online.
Types of analyses: For any purpose.
Mechanisms of data availability: Freely available.
Any additional restrictions: NA
